# Metabolic Reprogramming in the Opportunistic Yeast Candida albicans in Response to Hypoxia

**DOI:** 10.1128/mSphere.00913-19

**Published:** 2020-02-26

**Authors:** Anaïs Burgain, Faiza Tebbji, Inès Khemiri, Adnane Sellam

**Affiliations:** a CHU de Québec Research Center (CHUQ), Université Laval, Quebec City, Quebec, Canada; b Department of Microbiology, Infectious Diseases and Immunology, Faculty of Medicine, Université Laval, Quebec City, Quebec, Canada; University of Georgia

**Keywords:** *Candida albicans*, hypoxia, metabolomics

## Abstract

Hypoxia is the predominant condition that the human opportunistic fungus Candida albicans encounters in the majority of the colonized niches within the host. So far, the impact of such a condition on the overall metabolism of this important human-pathogenic yeast has not been investigated. Here, we have undertaken a time-resolved metabolomics analysis to uncover the metabolic landscape of fungal cells experiencing hypoxia. Our data showed a dynamic reprogramming of many fundamental metabolic pathways, such as glycolysis, the pentose phosphate pathway, and different metabolic routes related to fungal cell wall biogenesis. The C. albicans lipidome was highly affected by oxygen depletion, with an increased level of free fatty acids and biochemical intermediates of membrane lipids, including phospholipids, lysophospholipids, sphingolipids, and mevalonate. The depletion of oxygen-dependent lipids such as ergosterol or phosphatidylcholine with longer and polyunsaturated lateral fatty acid chains was observed only at the later hypoxic time point (180 min). Transcriptomics data supported the main metabolic response to hypoxia when matched to our metabolomic profiles. The hypoxic metabolome reflected different physiological alterations of the cell wall and plasma membrane of C. albicans under an oxygen-limiting environment that were confirmed by different approaches. This study provided a framework for future *in vivo* investigations to examine relevant hypoxic metabolic trajectories in fungal virulence and fitness within the host.

**IMPORTANCE** A critical aspect of cell fitness is the ability to sense and adapt to variations in oxygen levels in their local environment. Candida albicans is an opportunistic yeast that is the most prevalent human fungal pathogen. While hypoxia is the predominant condition that C. albicans encounters in most of its niches, its impact on fungal metabolism remains unexplored so far. Here, we provided a detailed landscape of the C. albicans metabolome that emphasized the importance of many metabolic routes for the adaptation of this yeast to oxygen depletion. The fungal hypoxic metabolome identified in this work provides a framework for future investigations to assess the contribution of relevant metabolic pathways in the fitness of C. albicans and other human eukaryotic pathogens with similar colonized human niches. As hypoxia is present at most of the fungal infection foci in the host, hypoxic metabolic pathways are thus an attractive target for antifungal therapy.

## Abstract (precis)

A critical aspect of cell fitness is the ability to sense and adapt to variations in oxygen levels in their local environment. Candida albicans is an opportunistic yeast that is the most prevalent human fungal pathogen. While hypoxia is the predominant condition that C. albicans encounters in most of its niches, its impact on fungal metabolism remains unexplored so far. Here, we provided a detailed landscape of the C. albicans metabolome that emphasized the importance of many metabolic routes for the adaptation of this yeast to oxygen depletion. The fungal hypoxic metabolome identified in this work provides a framework for future investigations to assess the contribution of relevant metabolic pathways in the fitness of C. albicans and other human eukaryotic pathogens with similar colonized human niches. As hypoxia is present at most of the fungal infection foci in the host, hypoxic metabolic pathways are thus an attractive target for antifungal therapy.

## INTRODUCTION

Candida albicans represents a major component of the human disease burden caused by fungi, and it is the most common cause of deadly invasive candidiasis (1). For a human-pathogenic fungus, metabolic flexibility is a critical virulence attribute that defines its ability to colonize different host niches with contrasting nutrient spectrums. C. albicans possesses a plastic metabolic machinery that promotes the efficient utilization of complex nutrient mixtures to sustain its fitness in the host (2). For instance, unlike the budding yeast Saccharomyces cerevisiae, C. albicans uses glycolysis, gluconeogenesis, and the glyoxylate cycle to assimilate concurrently glucose and other alternative carbon sources (3, 4). This specific evolutionary feature might contribute to an efficient utilization of complex combinations of carbon sources to promote fungal fitness in the different anatomical niches. Furthermore, inside the different human habitats, C. albicans also has to compete for nutrients with the local microbial cohabitants. Indeed, glucose is a growth-limiting carbon source when C. albicans grew in a mixed community of oral bacteria (5). C. albicans competes even with the host cells for glucose uptake, which subsequently leads to macrophage death and immune evasion (6). Therefore, both nutrient availability and competition have a significant impact on C. albicans fitness and pathogenicity inside the human host.

In addition to its higher metabolic versatility, C. albicans is also able to grow in environments with different oxygen concentrations (7). While C. albicans colonizes predominantly oxygen-poor niches, the impact of oxygen status in its primary metabolism was mostly neglected. Transcriptional profiling data have previously shown that in C. albicans and other human-pathogenic fungi, genes associated with oxygen-dependent metabolisms such as ergosterol, heme, and unsaturated fatty acids were upregulated as a compensatory response to the depletion of the aforementioned metabolites (8–13). Under hypoxic conditions, glycolytic genes are also activated, while those related to oxidative phosphorylation are repressed. As ATP levels were shown to drop when C. albicans experienced hypoxia (14), reactivation of glycolytic genes is most likely an adaptive response to compensate for such an energy leak. Genetic inactivation of the key glycolytic transcriptional activators Tye7 and Gal4 in C. albicans led to a substantial growth defect under hypoxia in addition to a significant alteration in virulence and the ability to colonize the gastrointestinal (GI) tract (15, 16). We have recently uncovered that the switch/sucrose nonfermentable (SWI/SNF) chromatin remodeling complex acts as a nexus for integrating oxygen status to the carbon metabolic machinery and fungal virulence (14). Taken together, these findings suggest that metabolic adaptation to oxygen depletion might be a deterministic feature of fungal biology and pathogenicity.

Low oxygen levels promote different C. albicans virulence attributes, including the invasive hyphal growth, biofilm formation, and chlamydospore development (7, 17). Furthermore, hypoxia influences positively, in favor of C. albicans, the outcome of host-pathogen interactions. Under microaerophilic environments, C. albicans cells reduce the exposition of their cell wall β-glucans as a strategy to conceal cell wall pathogen-associated molecular patterns (PAMPs) to mitigate recognition by detectin-1 and uptake by phagocytes (18, 19).

So far, the direct impact of hypoxia on the C. albicans metabolism remains completely unexplored. To fill this gap, we have undertaken temporal metabolomics profiling and provided a detailed metabolic landscape of fungal cells experiencing hypoxia. The hypoxic metabolome reflects different physiological alterations of C. albicans cells under an oxygen-limiting environment that were confirmed using different approaches. This study provided a framework for future *in vivo* investigations to examine relevant hypoxic metabolic routes in fungal virulence and fitness inside the host.

## RESULTS AND DISCUSSION

### Data overview of the metabolic response of C. albicans to hypoxia.

**FIG 1:**
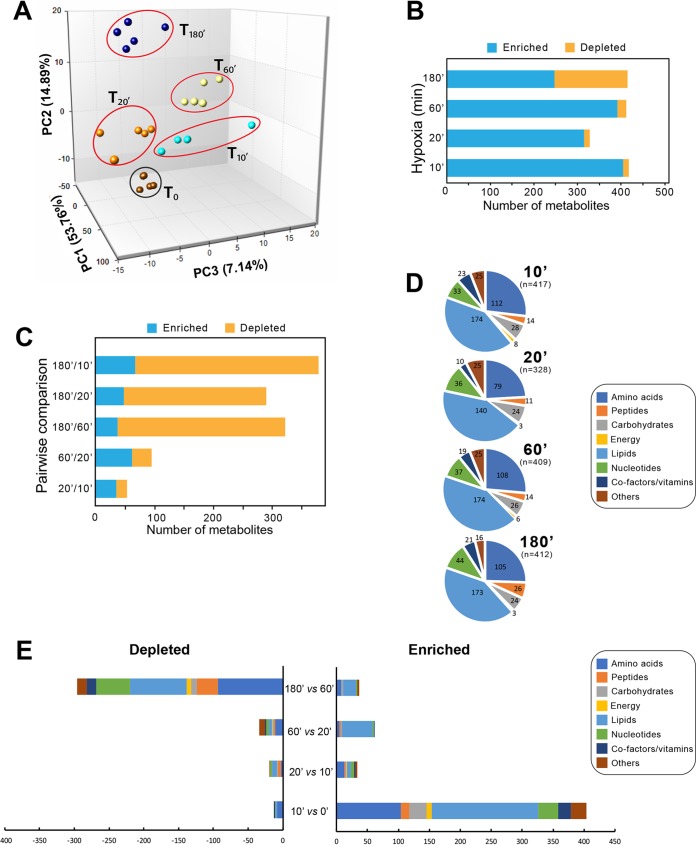
Hypoxia triggers significant changes in the C. albicans metabolome. (A) Principal-component analysis of the C. albicans hypoxic metabolome. Shown is a projection onto the three most explanatory principal components from biological replicates of each hypoxic time points. The percentage of the total variation that is encountered for each component is shown on the *x*, *y*, and *z* axes. (B) Number of metabolites that were either enriched or depleted across the hypoxic kinetic. Welch’s two-sample *t* test (*P* ≤ 0.05) was used to identify biochemicals that differed significantly in each hypoxic treatment. (C) Pairwise comparisons between hypoxic time points. Composition of metabolite classes (D) and metabolites enriched or depleted (E) at each hypoxic time point.

We performed temporal metabolomics profiling of the C. albicans SN250 strain growing as yeast on yeast extract-peptone-glucose (YPD) medium under hypoxic conditions (5% O_2_). Cells were harvested at 10, 20, 60, and 180 min post-hypoxia exposure and subjected to a detailed metabolomics analysis, where a total of 707 metabolites linked to different metabolic routes were detected by ultrahigh-performance liquid chromatography–tandem mass spectrometry (UPLC-MS/MS). Principal-component analysis (PCA) separated the metabolic signature of normoxic cells (T_0_) from those exposed to hypoxia, suggesting that C. albicans alters its metabolome to adapt to oxygen depletion (Fig. 1A and B; see also Table S1 in the supplemental material). These metabolic changes occurred not only between normoxic and hypoxic conditions but also between early (10 [T_10min_], 20 [T_20min_], and 60 min [T_60min_]) and late (T_180min_) exposure to hypoxia, as shown in the PCA plot. This was also reflected by the pairwise comparison of relative abundances of metabolites for each consecutive hypoxic time point (Fig. 1C). The 180-min exposure to hypoxia caused a significant change in 380, 290, and 332 metabolites compared to the earlier 10-, 20-, and 60-min time points, respectively. Of note, only 54 and 96 biochemicals were altered in the T_20min_/T_10min_ and T_60min_/T_20min_ pairwise comparisons, respectively.

10.1128/mSphere.00913-19.1 TABLE S1 Metabolites that were statistically altered during the exposure of C. albicans to hypoxia. The data set is presented as heat map, and it summarizes the numbers of biochemicals that achieved statistical significance (*P* ≤ 0.05), as well as those approaching significance (0.05 < *P* < 0.10). Download Table S1, XLSX file, 0.1 MB. Copyright © 2020 Burgain et al. 2020 Burgain et al. This content is distributed under the terms of the Creative Commons Attribution 4.0 International license.

Every class of metabolites was affected by oxygen depletion with a similar distribution across the hypoxic kinetic (Fig. 1D). However, in a comparison of both enriched and depleted metabolites specifying transitions between consecutive exposure times, we noticed that the major metabolic classes and their biochemical intermediates were enriched earlier at 10 min of hypoxia, while they were significantly depleted later at 180 min (Fig. 1E).

### Functional analysis of the metabolomic profiles.

**FIG 2:**
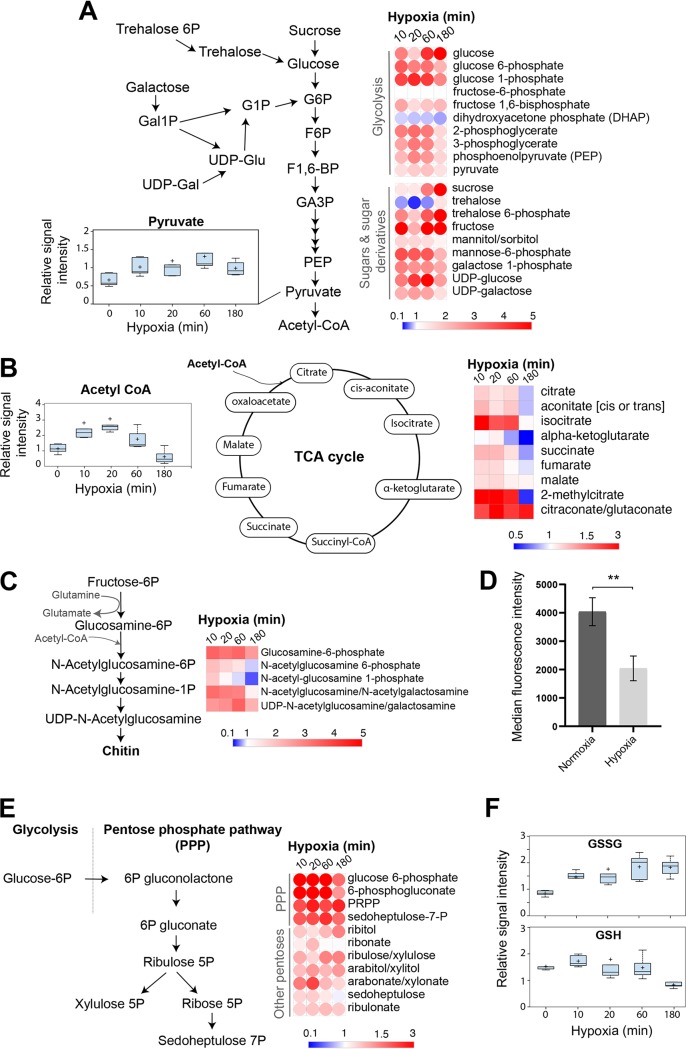
Effect of hypoxia on general carbon metabolism. Schematic of metabolisms associated with glycolysis (A), the TCA cycle (B), UDP-*N*-acetylglucosamine (C), and the pentose phosphate pathway (E) and the levels of their biochemical intermediates for each hypoxia time points. (D) Chitin quantification using CFW staining and fluorescence measurement in cells growing in YPD at 30°C in either normoxia or hypoxia. The results are the mean of the results from three biological replicates. (F) Redox status of hypoxic C. albicans cells as reflected by the level of oxidized (GSSG) and reduced (GSH) glutathione. G6P, glucose-6-phosphate; G1P, glucose-1-phosphate; F6P, fructose-6-phosphate; F1,6-BP, fructose-1,6-bisphosphate; GA3P, glyceraldehyde-3-phosphate; PEP, phosphoenolpyruvate; Gal1P, galactose-1-phosphate; UDP-Glu, UDP-glucose; UDP-Gal, UDP-galactose; PRPP, 5-phosphoribosyl diphosphate.

**FIG 3:**
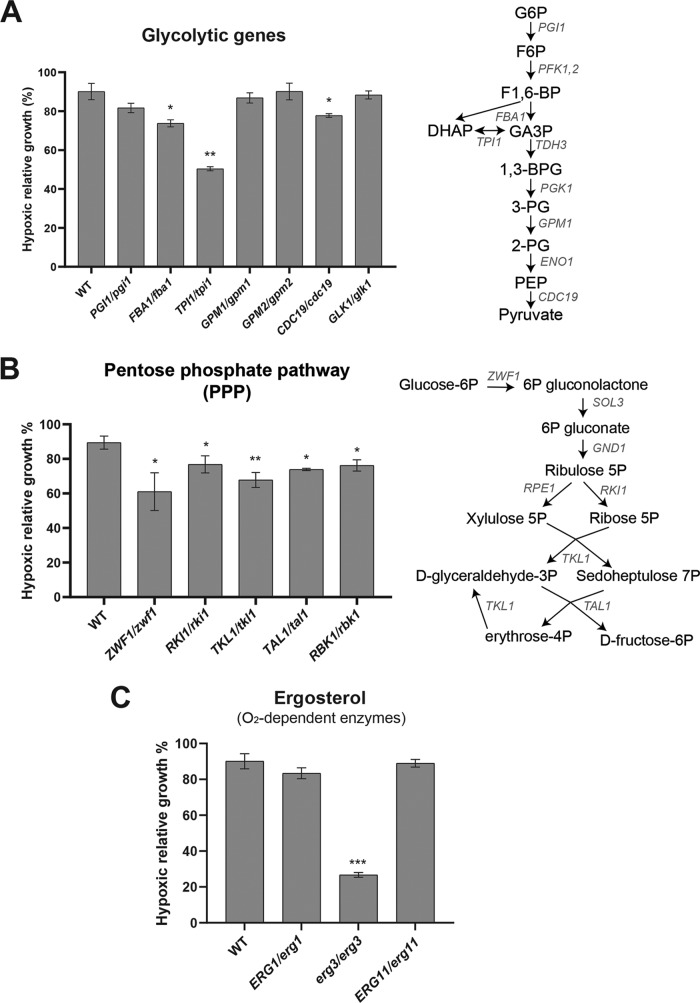
Effect of genetic perturbation of glycolysis, pentose phosphate pathway, and ergosterol metabolism on C. albicans survival under hypoxia. (A and B) Haploinsufficiency of the glycolytic (A) and the pentose phosphate pathway (B) genes for growth under hypoxia. (C) Survival of the heterozygous (*erg11*/*ERG11* and *erg1*/*ERG1*) and the homozygous (*erg3*) oxygen-dependent ergosterol biosynthetic genes under hypoxia. Mutants and the parental wild-type (WT) (CAI4) strains were grown in SC medium at 30°C under both normoxic (21% oxygen) and hypoxic conditions (5% oxygen) for 24 h. The hypoxic relative growth was determined as the OD ratio of the hypoxic to the normoxic cultures and is expressed as a percentage. Heterozygous mutants were considered for these assays because their corresponding genes are essential. The results are the means of the results from at least three biological replicates. Statistical significance was tested using Student's *t* test and is indicated as follows: *, *P* < 0.05; **, *P* < 0.01; ***, *P* < 0.001.

**(i) Carbohydrates.** As a eukaryotic organism, C. albicans metabolizes glucose to pyruvate via glycolysis. However, in opposition to S. cerevisiae, C. albicans is a Crabtree-negative organism that oxidizes pyruvate to carbon dioxide and H_2_O through the tricarboxylic acid (TCA) cycle under normoxia and uses fermentation to convert pyruvate to ethanol and CO_2_ under hypoxic or anoxic environments (16, 20). Previous investigations showed that in response to oxygen depletion, C. albicans and many other pathogenic fungi activate glycolytic genes and repress those associated with the TCA cycle (10, 21). Accordingly, our metabolomic analysis showed increased levels of all glycolytic intermediates and pyruvate (Fig. 2A). Acetyl-coenzyme A (acetyl-CoA) and TCA cycle intermediates increased at early exposure to hypoxia and dropped significantly at 180 min (Fig. 2B). As many glycolytic intermediates are substrates for amino acids, nucleotides, and lipid biosynthesis, our data suggest that glycolysis was refueled to fulfill the macromolecular demands in C. albicans cells experiencing hypoxia. Alternatively, metabolic reactivation of glycolysis might also compensate for the ATP depletion as a result of the reduction in respiration activity that accompanies hypoxia, as we have previously shown (14). As most of the glycolytic genes are essential (22), we assessed the growth of different heterozygous mutants under hypoxia. The triose-phosphate isomerase *TPI1* gene, the fructose-bisphosphate aldolase *FBA1* gene, and the pyruvate kinase *CDC19* gene were haploinsufficient under hypoxia compared to normoxia, emphasizing the critical role of glycolysis for the hypoxic metabolic reprogramming in C. albicans (Fig. 3A).

Other sugar derivatives, such as activated nucleotide hexoses (UDP-glucose and UDP-galactose), fructose, and polyols (sorbitol or mannitol), were also increased under the different hypoxic time points, suggesting that glucose fluxes into other alternative metabolic routes (Fig. 2A). UDP-sugars act as glycoconjugates for glycosyltransferase reactions to generate cell wall or membrane glycoproteins and are also precursors of structural polysaccharides such as glucans. The accumulation of UDP-glucose and UDP-galactose might thus reflect an impaired glucan biosynthesis and, consequently, a perturbation of the C. albicans cell wall integrity.

At 10 to 60 min of hypoxia, the levels of all chitin metabolic intermediates were increased (Fig. 2C). The sustained increase of the key building block of chitin, UDP-*N*-acetylglucosamine, across all hypoxic time points mirrors a reduction in metabolic support for chitin biosynthesis. Using calcofluor white (CFW) staining, we found that the chitin content was significantly reduced in C. albicans cells experiencing hypoxia compared to normoxic cells (Fig. 2D).

The pentose phosphate pathway (PPP) is an essential component of cellular metabolism that provides precursors for nucleotide and amino acid biosynthesis. The oxidative branch of the PPP produces the reducing NADPH which serves as a cofactor for many anabolic enzymes and maintains redox balance under oxidative stress (23). As for the glycolytic metabolites, intermediates of the PPP exhibited increased levels in C. albicans cells under hypoxia (Fig. 2E). This metabolic signature reflects an enhanced shunting into either nucleotide or amino acid anabolic pathways or a supply of the hypoxic fungal cells with NADPH to prevent oxidative stress and maintain redox homeostasis. NADPH is an essential cofactor for the glutathione-dependent enzymes that protect cells against oxidative damage, such as the glutathione reductases that reduce glutathione disulfide (GSSG) to the sulfhydryl form of glutathione (GSH). Indeed, our data showed an accumulation of GSSG and a stable amount of GSH that are reminiscent of oxidative stress (Fig. 2F). Different heterozygous mutants of the PPP exhibited a significant growth reduction under hypoxia, stressing the role of this essential pathway in fungal hypoxic adaptation (Fig. 3B).

**FIG 4:**
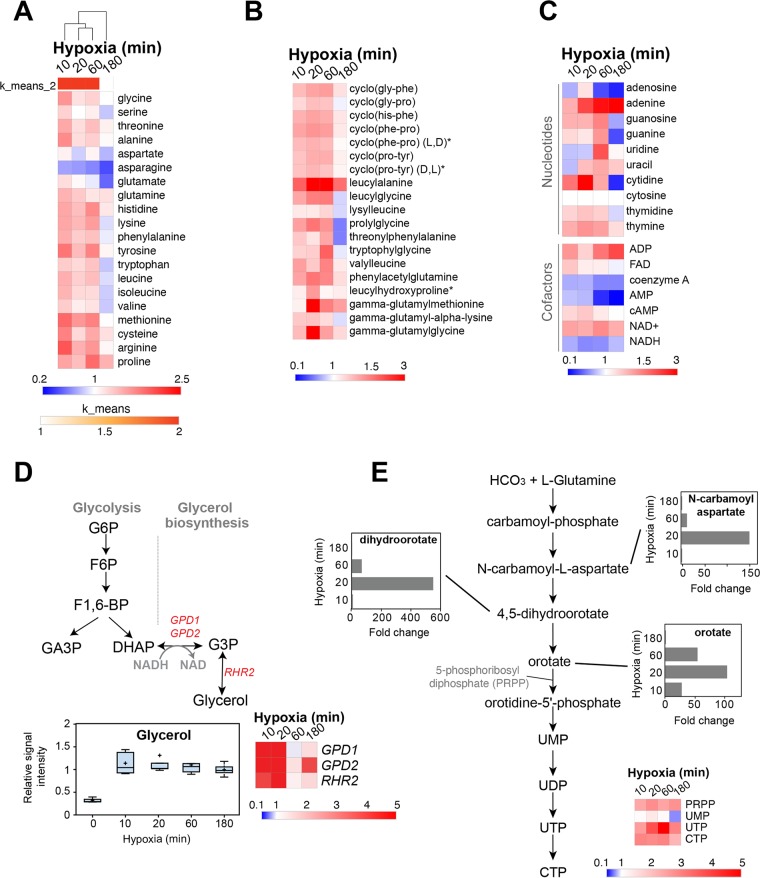
Effect of hypoxia on amino acid, peptide, and nucleotide metabolisms. (A) Clustergram of amino acid fold changes under hypoxia compared to normoxia. K-means clustering analysis separates the early hypoxic treatments (10 to 60 min) from the late hypoxic exposure (180 min) regarding amino acid level changes. (B and C) Abundance of different species of dipeptides (B) and nucleotide and nucleotide derivative cofactors (C) for each hypoxia time point. FAD, flavin adenine dinucleotide. (D) Accumulation of glycerol upon exposure of C. albicans cells to hypoxia. Transcript levels of glycerol biosynthetic genes, including the glycerol-3-phosphate dehydrogenase genes *GPD1* and *GPD2* in addition to the glycerol 3-phosphatase *RHR2* from previously published (10) and current (Table S2) investigations are shown. G6P, glucose-6-phosphate; F6P, fructose-6-phosphate; F1,6-BP, fructose-1,6-bisphosphate; GA3P, glyceraldehyde-3-phosphate; DHAP, dihydroxyacetone phosphate; G3P, glycerol-3-phosphate. (E) Schematic of the pyrimidine ribonucleotide *de novo* biosynthesis pathway. The levels of *N*-carbamoylaspartate, dihydroorotate, and orotate intermediates under hypoxic conditions are shown as histograms. Relative levels of pyrimidine nucleoside phosphates (UMP, UTP, and CTP) and 5-phosphoribosyl diphosphate (PRPP) across the hypoxic time course are also displayed as a heat map.

**(ii) Amino acids.** Generally, the amounts of most of the amino acids were increased when cells were exposed to early hypoxic treatments (10 to 60 min) and declined after 180 min (Fig. 4A). Of note, asparagine (Asn) levels were reduced during the whole hypoxic treatments. As a gluconeogenic amino acid, Asn might be used to fuel glycolysis by its conversion to phosphoenolpyruvate (PEP). The amounts of different dipeptides were increased during the early exposure to hypoxia, suggesting an increased catabolism of proteins to enrich the pool of free amino acids in C. albicans cells (Fig. 4B).

10.1128/mSphere.00913-19.2 TABLE S2 Differentially expressed transcript during the hypoxic time course. Download Table S2, XLSX file, 0.6 MB. Copyright © 2020 Burgain et al. 2020 Burgain et al. This content is distributed under the terms of the Creative Commons Attribution 4.0 International license.

**(iii) Nucleotides and their derivatives.** Overall, the abundance of nucleotides follows the same trends as amino acids (Fig. 4C), in which most nucleotides accumulated earlier and decreased at 180 min of hypoxia. ADP and cyclic AMP (cAMP) levels were increased, whereas AMP and coenzyme A were significantly depleted in hypoxic fungal cells.

The reduced form of NAD (NADH) is produced during glycolysis, and it is reoxidized through the respiratory chain. Under hypoxia, as glucose fluxes into glycolysis and respiration is reduced, we might expect the NADH/NAD ratio to be increased. Intriguingly, our metabolomic analysis supports an opposite tendency since the NAD^+^ form was increased while NADH was depleted. One explanation for this finding is that NADH is overoxidized to NAD through the glycerol biosynthesis route, a fact supported by a significant increased accumulation of glycerol, as well as the activation of glycerol biosynthetic transcripts along the hypoxic kinetic (Fig. 4D).

*N*-Carbamoylaspartate, dihydroorotate, and orotate, which are intermediates of the pyrimidine ribonucleotide *de novo* biosynthesis pathway, were the highly abundant metabolites, and they were transiently formed in response to the early exposure hypoxia (10 to 60 min) (Fig. 4E). Intriguingly, the accumulation of these three metabolites does not correlate with the amplitude of the increase of the downstream intermediates or even the final nucleotide products (UTP and CTP) (Fig. 4E). This phenomenon is similar to what was reported in mammalian cells, where the glutamine nitrogen was enriched in excreted dihydroorotate and orotate rather than processing to its downstream UMP under hypoxia as an alternative strategy to dispose of ammonia (24).

**FIG 5:**
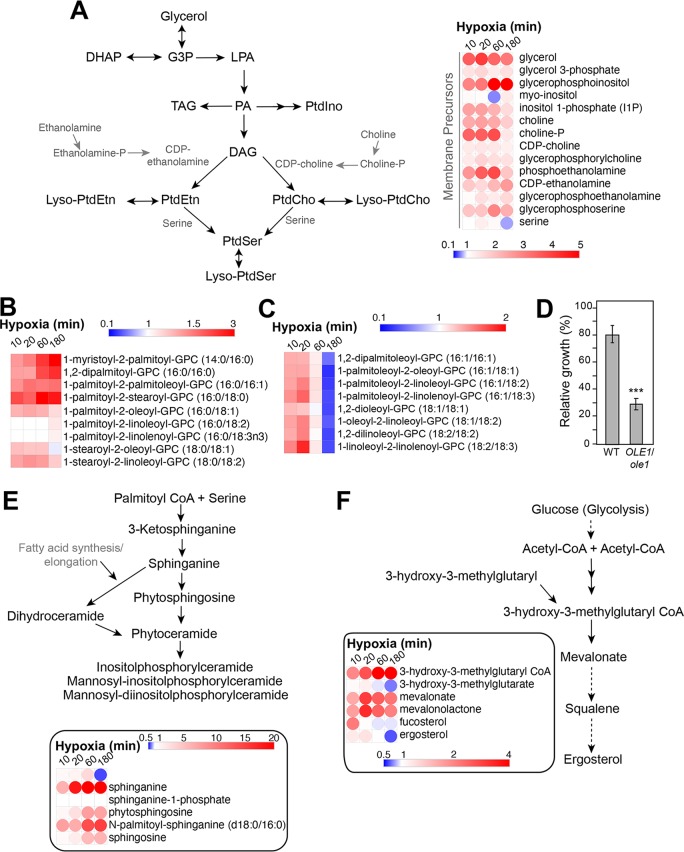
Hypoxia alters the C. albicans lipidome. (A) Annotated pathway schematics for glycerophospholipids and their levels in response to hypoxia. DHAP, dihydroxyacetone phosphate; G3P, glycerol-3-phosphate; LPA, lysophosphatidic acid; PA, phosphatidic acid; TAG, triacylglycerol; DAG, diacylglycerol; PtdIno, phosphatidylinositol; PtdEtn, phosphatidylethanolamine; PtdSer, phosphatidylserine; PtdCho, phosphatidylcholine. (B and C) Differential effects of hypoxia on the level of phosphatidylcholines (PtdCho) with 14- to 18-carbon-length fatty acids and reduced unsaturation (B) and those with polyunsaturation in one chain of more than 20 carbons (C). (D) The fatty acid desaturase gene *OLE1* is haploinsufficient for hypoxic growth. WT (CAI4) and an *ole1*/*OLE1* heterozygous mutant were grown on SC in both normoxia and hypoxia under agitation at 30°C for 48 h. For each strain, results represent the mean growth under hypoxia relative to normoxia of at least three replicates. Statistical significance was tested using Student's *t* test (***, *P* < 0.001). (E and F) Hypoxia alters the metabolism of sphingolipids (E) and ergosterol (F).

**(iv) Lipids.** Across all hypoxic kinetics, C. albicans exhibited an increase in free fatty acids (FFA) regardless of their chain length and degree of unsaturation (Table S1). The same trend was also noticed for other specific lipid classes, including phospholipids, lysophospholipids, sphingolipids, mevalonate, and acylglycerol lipids (mono- and diacylglycerol) (Table S1 and Fig. 5A, C, E, and F). The elevated FFA together with the depletion of acetyl-CoA (Fig. 2B and 5B and C) suggest that under hypoxia, C. albicans reduces the oxidation of long-chain FFA. We also found that the majority of the detected glycerophospholipids, including phosphatidylethanolamine (PtdEtn), phosphatidylserine (PtdSer), and phosphatidylinositol (PtdIno), as well as their precursors (choline, inositol, and ethanolamine), were increased at 10 to 60 min of hypoxia and significantly dropped at 180 min (Table S1 and Fig. 5A).

Phosphatidylcholine (PtdCho), with 14- to 18-carbon-length FAs and unsaturation in only one of the two lateral FA chains, were enriched across all hypoxia kinetics. However, PtdCho with unsaturation in both FA chains or with polyunsaturation in one chain of more than 20 carbons were increased at 10 to 60 min of hypoxia and declined at 180 min (Fig. 5B and C). This disparity can be explained by the fact that oxygen is required for fatty acid desaturation that becomes a rate-limiting process under hypoxia. In agreement with that, we showed that the essential fatty acid desaturase gene *OLE1* in C. albicans was haploinsufficient under hypoxia (Fig. 5D).

While mevalonate was increased across the hypoxic kinetics, ergosterol was significantly depleted at 180 min of hypoxia (Fig. 5F). Given the oxygen dependency of ergosterol biosynthesis, it is thus expected that this fungal sterol dropped under hypoxia. It is even thought that ergosterol depletion is a cellular cue in C. albicans to sense and adapt to oxygen depletion (9). However, as shown by the current metabolomic investigation and our previous transcriptional studies (10), depletion of ergosterol occurred only after 60 min of exposure to hypoxia, which rules out the fact that the depletion of this metabolite is not a rapid oxygen-sensing mechanism. Our genetic inactivation experiment of the oxygen-dependent ergosterol biosynthetic genes in C. albicans (*ERG1*, *ERG3*, and *ERG11*) underlined an essential role of the C-5 sterol desaturase Erg3 for hypoxic growth (Fig. 3C). Taken together, the lipidome analysis reflects a drastic change in FFA and membrane lipid metabolisms and supports a remodeling of the plasma membrane of the fungal cells in response to hypoxia.

### Transcriptional profiling data corroborate the main metabolic adaptation to hypoxia.

**FIG 6:**
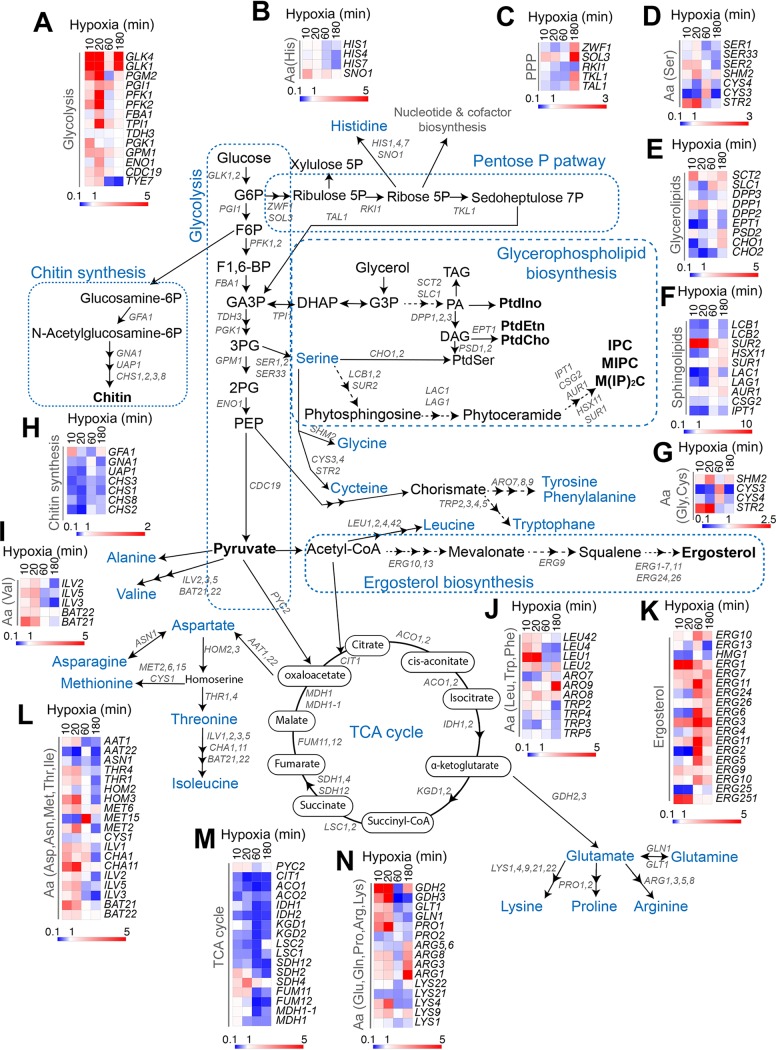
Correlation analysis of the hypoxic transcriptome and metabolome data. The different altered oxygen-sensitive metabolic routes and the dynamic of transcripts that were significantly altered during the corresponding time points are summarized. (A to N) Differential expression of metabolic genes associated with glycolysis (A), amino acid biosynthesis (histidine [B] serine [D], glycine and cysteine [G], valine [I], leucine, tryptophan, and phenylalanine [J]; aspartate, asparagine, methionine, threonine, and isoleucine [L], and glutamine, glutamate, proline, arginine, and lysine [N]), the pentose phosphate pathway (C), lipids (glycerolipids [E], sphingolipids [F], ergosterol [K]), chitin synthesis (H), and the TCA cycle (M) are shown as clustergrams. Upregulated and downregulated genes are indicated by red and blue, respectively. The 10- to 60-min transcriptional data were extracted from a study by Sellam et al. (10). The full list of differentially expressed transcripts at 180 min of hypoxia is available in Table S2. Aa, amino acid.

To assess whether transcription is concomitantly altered with the C. albicans metabolome in response to hypoxia, we have matched the main observed metabolic signatures to gene expression profiling. We have previously provided a comprehensive transcriptional profiling of C. albicans cells experiencing hypoxia over an early time course up to 60 min (comprising 10-, 20-, and 60-min hypoxic exposures) (10). In the current work, we have extended this analysis to include the 180-min time point. Figure 6 summarizes the main metabolic changes and the dynamics of transcripts that were significantly altered during the corresponding time points.

**(i) Carbohydrate.** Transcripts of glycolytic genes were activated, while those of TCA cycle were downregulated following the same alteration of their corresponding metabolites (Fig. 6A and M). While PPP intermediates accumulated across all the hypoxic time points, induction of PPP transcripts was perceived only after 180 min of hypoxia (Fig. 6C). UDP-*N*-acetylglucosamine biosynthetic and chitin polymerization genes were downregulated in all hypoxic treatments (Fig. 6H). Indeed, the accumulation of UDP-*N*-acetylglucosamine without being efficiently polymerized to form chitin might have a negative feedback into the transcriptional control of chitin biosynthesis.

**(ii) Lipids.** Transcript levels of ergosterol metabolic genes were increased at 60 to 180 min of hypoxia, most likely as a compensatory response to the decreased content of this fungal sterol, as shown above (Fig. 6K). A similar transcriptional behavior of *ERG* genes was previously reported when C. albicans grew under sterol-depleted conditions (25). Transcripts enriched in glycerolipid metabolism were overall induced at 60 to 80 min of oxygen scarcity (Fig. 6E). Of note, the genes involved in the conversion of glycerol-3-phosphate (G3P) to phosphatidic acid (PA), including *SCT2* and *DPP1*, were induced across all of the hypoxic time course, which corroborates the increased levels of different classes of glycerophospholipids (Table S1).

**(iii) Amino acids.** Generally, as for most of amino acids and their intermediates, most of their corresponding transcripts were induced early at 10 to 20 min of hypoxia and repressed at 60 to 180 min (Fig. 6B, D, G, I, J, and L). The exception to this trend was the induction of arginine biosynthetic genes *ARG1*, *ARG2*, *ARG8*, and *ARG5*,*6* that were highly upregulated at 180 min (Fig. 6N). This induction might reflect an attempt of C. albicans cells to supplement *de novo* the cellular content of arginine, as many transcripts of different nitrogen compounds and amino acids permeases (*CAN2*, *MEP1*, *GAP6*, and *DUR4*) were induced (Table S2).

### The hypoxic metabolome reflects different physiological alterations of C. albicans cells under an oxygen-limiting environment.

**FIG 7:**
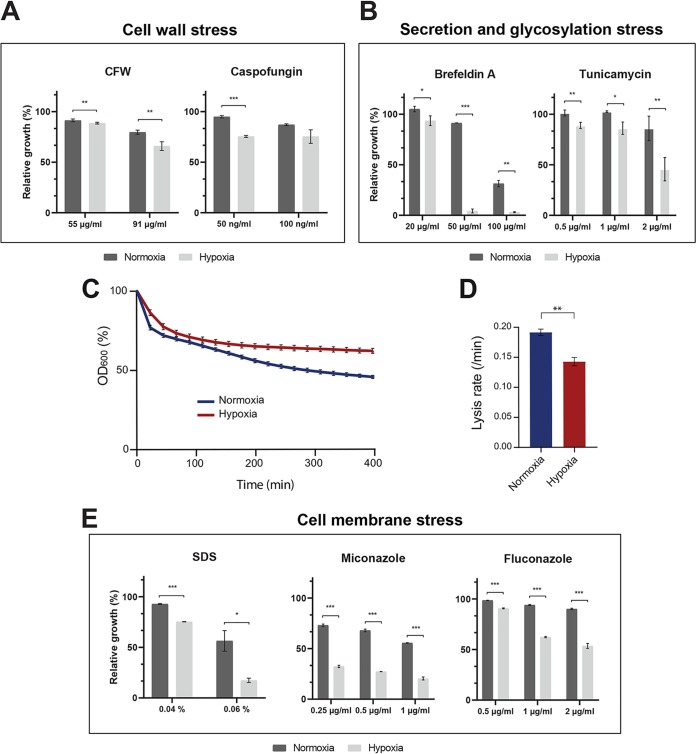
The hypoxic metabolome reflects different physiological alterations of C. albicans cells. (A to E) Hypoxia sensitizes C. albicans cells to cell wall (A, C, and D), secretion (B), and cell membrane (E) stressors. Cells were grown in YPD medium at 30°C with the corresponding inhibitors under both normoxic (21% oxygen) and hypoxic (5% oxygen) conditions for 24 h. The hypoxic relative growth (A, B, and E) was determined as the OD ratio of the treated to the nontreated cultures under both normoxia and hypoxia and is expressed as a percentage. The results are the means of the results from at least three biological replicates. Statistical significance was tested using Student's *t* test and is indicated as follows: *, *P* < 0.05; **, *P* < 0.01; ***, *P* < 0.001. (C) Indirect quantification of β-glucan masking using the β-glucanase digestion assay. Cell lysis as represented by the OD_600_ decrease reflects an increased β-1,3-glucanase activity which is proportional to cell wall β-glucan exposure. (D) The initial rate of cell lysis was calculated from the first 250 min after the addition of β-glucanase. The results are the means of the results from three biological replicates. Statistical significance was tested using Student's *t* test (**, *P* < 0.01).

Our metabolomic profiling underscored distinctive metabolic signatures that might mirror different cellular alterations as a consequence of oxygen depletion. The accumulation of UDP-sugars in hypoxic cells might reflect a defect in glucan synthesis or glycosylation activity. C. albicans hypoxic cells exposed to the cell wall-perturbing agents caspofungin and CFW exhibited a significant growth reduction compared to normoxic cells (Fig. 7A). A similar growth defect was observed when cells were treated with the glycosylation inhibitor tunicamycin or the endoplasmic reticulum (ER)-to-Golgi inhibitor brefeldin A, where most of glycosyltransferase reactions take place (Fig. 7B). As shown for chitin, our results suggest that hypoxia perturbs the metabolism of different cell wall building blocks, which might affect the integrity of this essential fungal organelle. Accordingly, previous studies have shown that hypoxia leads to β-glucan reduction and masking in C. albicans, which in turn leads to escape from the innate immune surveillance and increased virulence (19, 20). To measure β-glucan exposure, we tested the sensitivities of both normoxic and hypoxic C. albicans cells to the recombinant β-glucanase (26). Cells exposed to normoxia had increased sensitivity to β-glucanase compared to that with hypoxic cells, supporting the idea that low oxygen levels promote β-glucan masking in C. albicans (Fig. 7C and D). Thus, reprogramming cell wall metabolism in an oxygen-depleted environment should confer a fitness advantage to C. albicans by hiding from the immune system to sustain commensalism in the host.

Alterations in the levels of different class of lipids in addition to the requirement of key ergosterol biosynthetic genes for the hypoxic growth reflect a rearrangement of the C. albicans cell membrane under an oxygen-deprived environment. C. albicans exhibited an augmented sensitivity to different cell membrane stressors, such as SDS or ergosterol inhibitors (fluconazole and miconazole), when grown under hypoxia compared to normoxic conditions (Fig. 7E). Taken together, our data reflect that hypoxia induces an important remodeling in the metabolic routes of many cell membrane and cell wall building blocks, which consequently lead to physiological alteration of the aforementioned organelles.

### Conclusion.

Exposure of C. albicans to hypoxia had a significant influence on the metabolome and the lipidome of this opportunistic yeast. Our investigation provided a detailed metabolic landscape of fungal cells experiencing hypoxia, with many differentially activated and intertwined metabolic routes that were critical for C. albicans survival under hypoxia. Furthermore, our metabolomics and transcriptomics analyses allowed us to probe biological and metabolic networks which are a rich starting point for future assessment for their role in fungal physiology and survival in the host. A microorganism metabolome is highly labile and might depend on conditions that are highly dynamic in the human host, such as oxygen level, temperature, nutrient availability, stress, and inoculum size (cell density). At the later hypoxic time point (180 min), the expressed metabolome might illustrate, in addition to hypoxia, the effects of increased cell density and nutrient deprivation. In this regard, this work serves also an entry point to narrow down and to confirm specific hypoxic metabolic routes and their dynamics using, for instance, a fluxomics approach under a myriad of combined conditions mimicking the host environment.

## MATERIALS AND METHODS

### Growth conditions and strains.

For general propagation and maintenance conditions, cells were cultured at 30°C in YPD medium (10 g l^−1^ yeast extract, 20 g l^−1^ peptone, 20 g l^−1^ glucose) supplemented with 50 mg/liter uridine in a rotary shaker. The strains used in this study are listed in Table S3.

10.1128/mSphere.00913-19.3 TABLE S3 C. albicans strains used in this study. Download Table S3, XLSX file, 0.1 MB. Copyright © 2020 Burgain et al. 2020 Burgain et al. This content is distributed under the terms of the Creative Commons Attribution 4.0 International license.

### Metabolomics analysis.

C. albicans strain SN250 was cultured in 150 ml of YPD at 30°C in a rotary shaker overnight. The cells were centrifuged, resuspended in 150 ml of fresh YPD medium, and grown at 30°C to an optical density at 600 nm (OD_600_) of 0.8. A fraction of cells was frozen in liquid nitrogen and corresponds to the normoxic condition (T = 0). The remaining cell suspension was added to an OD_600_ of 1 into hypoxic bottles containing 45 ml of fresh YPD medium flushed with nitrogen to remove oxygen (5% oxygen). Cultures were incubated at different times (10, 20, 60, and 180 min). Hypoxic bottles were opened in an anaerobic chamber, where cultures were transferred to a centrifuge tube. After centrifugation at 2,000 × *g* for 2 min, cell pellets were immediately frozen in liquid nitrogen. For each condition, a total of five replicates were analyzed. Metabolomics analysis was carried out in collaboration with Metabolon (Durham, NC, USA). To remove protein, dissociate small molecules bound to protein, and recover chemically diverse metabolites, proteins were precipitated with methanol under vigorous shaking for 2 min, followed by centrifugation. The resulting extracts were analyzed by gas chromatography-mass spectrometry (GC-MS) and liquid chromatography-mass spectrometry (LC-MS). The amounts of each metabolite were normalized against the total protein levels in each sample, as determined by a Bradford assay. All metabolites with mean values that had significant differences (*P* < 0.05) between treated and untreated samples were considered enriched (>1.5-fold) or depleted (<1.5-fold).

### Growth and drug susceptibility assays.

Overnight cultures of different mutants were washed twice with phosphate-buffered saline (PBS) buffer and adjusted to an OD_600_ of 0.01 in SC medium (1.7 g l^−1^ yeast nitrogen base without amino acid and ammonium, 20 g l^−1^ glucose, 2 g l^−1^ amino acid mix, 5 g l^−1^ ammonium sulfate) supplemented with 50 mg/liter uridine. For the tetracycline repressor gene replacement and conditional expression (GRACE) mutants, tetracycline was added to the medium at a concentration of 100 μg/ml. Growth was assessed in a 96-well plate using a Cytation 5 plate reader at 30°C under normoxic (21% oxygen) or hypoxic (5% oxygen) conditions, with OD_600_ readings taken every 10 min for 48 h. The hypoxic relative growth was determined as the OD ratio of the hypoxic to the normoxic cultures and is expressed as a percentage.

For drug susceptibility and inhibitor assays, overnight cultures were washed twice in PBS, and their OD_600_ was adjusted to 0.01 in YPD. A total volume of 98 μl of fungal cells was added to each well of a 96-well plate in addition to 2 μl of the corresponding concentration of the inhibitor. Growth was assessed using a Cytation 5 plate reader at 30°C under normoxic or hypoxic (5% oxygen) conditions.

### Chitin quantification.

Mid-log-phase SN250 cells were collected by centrifugation, washed twice with PBS, and resuspended in YPD. Half of the cell suspension was used to inoculate aerated flasks containing 20 ml of fresh YPD (normoxia), and the second half was added to the bottles containing fresh YPD medium flushed with nitrogen to remove oxygen (hypoxia). After incubation for 3 h at 30°C, cells were centrifuged, fixed with 1 ml of paraformaldehyde 4% solution for 1 h at 30°C, and then washed twice with PBS. The yeast concentration was adjusted to 1 × 10^6^ cells/ml in 500 μl of sterile water and stained with 2.5 μg ml^−1^ CFW for 15 min at room temperature. Blue fluorescence (4′,6-diamidino-2-phenylindole [DAPI] channel) emitted by 50,000 cells was quantified using a BD LSR II SORP cytometer. The median fluorescence intensity represents the fluorescence emitted from stained cells (positive population) minus the fluorescence emitted from unstained cells (autofluorescence).

### β-Glucanase sensitivity assay.

The β-glucanase assay was performed as previously described (26). Yeast cells were grown under the same conditions as for the chitin content assay. After 3 h of incubation under normoxia or hypoxia, cells were washed twice with sterile water and resuspended in the assay buffer (40 mM 2-mercaptoethanol, 50 mM Tris-HCl [pH 7]) at an OD_600_ of 1, and a total of 190 μl was added to triplicate wells of a 96-well plate. β-1,3-Glucanase (Sigma-Aldrich) was resuspended in sterile water to 2 U/ml, and 10 μl was added to each well. The OD_600_ was measured every 20 min at 30°C with a Cytation 5 plate reader as a measure of cell lysis, and data are expressed as a percentage of the OD_600_ at the initial time point. The rate of cell lysis was determined from the first 250 min.

### Expression analysis by microarrays.

DNA microarray experiments were performed as for the metabolomics profiling, as previously described (10). Briefly, overnight cultures of strain SN250 were diluted to an OD_600_ of 0.1 in 100 ml of fresh YPD-uridine medium, grown at 30°C to an OD_600_ of 0.8. Half of the C. albicans cell suspension was used to inoculate aerated flasks containing fresh YPD medium (normoxia), and the second half was added to bottles containing fresh YPD medium flushed with nitrogen to remove oxygen (hypoxia). Cultures were then incubated for 180 min, and cells were then harvested by centrifugation. Two biological replicates were performed. Total RNA was extracted using an RNeasy purification kit (Qiagen) and glass bead lysis in a Biospec Mini bead-beater. Total RNA was eluted and assessed for integrity on an Agilent 2100 Bioanalyzer prior to cDNA labeling, microarray hybridization, and analysis (27). Differentially expressed transcripts shown in Table S2 were identified using Welch’s *t* test, with a false-discovery rate (FDR) of 5% and 1.5-fold enrichment cutoff.

### Data availability.

Normalized gene expression and metabolomics (Metabolon) data are publicly available in the supplemental material.

10.1128/mSphere.00913-19.4 TABLE S4 Raw data of the time-resolved hypoxic metabolome. Download Table S4, XLSX file, 0.3 MB. Copyright © 2020 Burgain et al. 2020 Burgain et al. This content is distributed under the terms of the Creative Commons Attribution 4.0 International license.
